# Failure of Conflict to Modulate Central Executive Network Activity Associated with Delusions in Schizophrenia

**DOI:** 10.3389/fpsyt.2013.00113

**Published:** 2013-09-23

**Authors:** William J. Speechley, Todd S. Woodward, Elton T. Ngan

**Affiliations:** ^1^Department of Psychiatry, Schizophrenia Cognition Imaging Laboratory, University of British Columbia, Vancouver, BC, Canada; ^2^Department of Psychiatry, Cognitive Neuroscience of Schizophrenia Laboratory, University of British Columbia, Vancouver, BC, Canada

**Keywords:** delusions, schizophrenia, cognition, decision-making

## Abstract

Dual-stream modulation failure (DSMF) proposes that dysfunctional regulation of logical and intuitive decision-making processes by conflict and emotional salience may be the underlying cognitive mechanism for the formation and maintenance of delusions in schizophrenia. The present study utilizes a combination of emotionally salient and neutral stimuli in conflict and non-conflict conditions in a sentence verification task to test specific hypotheses predicted by the model. Twenty-one patients with schizophrenia and 21 controls completed a sentence verification task with fMRI acquisition. The results are consistent with the predictions based on the conflict modulation component of the model, but do not support the emotional modulation component of the model.

## Introduction

The Diagnostic and Statistical Manual of Mental Disorders (1) defines delusions as false beliefs based on incorrect inferences about external reality that are firmly sustained despite what almost everybody else believes and despite what constitutes incontrovertible and obvious proof or evidence to the contrary. Delusions are one of the cardinal, and most debilitating symptoms of schizophrenia. While there have been improvements in treatment compliance due to the improved tolerability of atypical neuroleptics, medications remain partially effective for some and completely ineffective for others. Further improvements in the management of schizophrenia will require further advances in our understanding of the neuropathophysiology of psychosis. The rapid development of neuroimaging technologies and new models of human decision-making across the last decade have provided an opportunity to advance our understanding of the cognitive and neuropathophysiological basis for psychosis in general and delusions in particular.

A number of cognitive models have been developed offering accounts of the emergence and subsequent persistence of delusions in schizophrenia and other psychiatric illnesses. Frith (2, 3) has suggested that a Theory of Mind deficit may underlie the formation of delusions of reference and persecution. In short, the inability to adequately discern the thoughts, beliefs, and intentions of others may lead to the misidentification of malevolence where none exists. Bentall has suggested that an attributional bias may act as a self-esteem defense mechanism contributing to the formation and maintenance of persecutory delusions (4, 5). Here, an individual maintains self-esteem by minimizing contradictions between the “actual self” and the “ideal self” by holding others responsible for negative events (i.e., externalizing and personalizing) and taking credit for positive events (i.e., internalizing). While the Theory of Mind and Attributional Bias accounts have good explanatory power for persecutory delusions, and provide an explanation for the content of these sorts of delusions, they are specific to persecutory delusions, rather than providing more general models of delusions.

The jumping-to-conclusions (JTC) bias (6, 7) and the bias against disconfirmatory evidence [BADE; (8)] are two complementary models that offer more general theories of delusions. JTC describes an aberration in probabilistic reasoning that may contribute to the formation of delusional beliefs. Specifically, arbitrary inference (9) and a lack of active reality testing (10) may limit the extent to which an individual seeks out and assesses data, resulting in firm decisions being made relatively sooner and on the basis less evidence than typically seen in healthy controls. Thus, erroneous beliefs may be readily accepted with minimal evidential support. BADE can be considered a delusion maintenance model, highlighting a bias away from evidence that challenges an existing belief. BADE experiments require the evaluation and re-evaluation of scenarios or pictures as progressively more explanatory evidence is provided. These studies have consistently shown that, compared to healthy controls, people with schizophrenia (11, 12) make less adjustment to their beliefs when confronted with evidence that should result in belief re-evaluation. Some research has suggested that this effect may be stronger for currently delusional people (8, 13).

The results for JTC and BADE have proven robust, and have been identified using a number of variations on the original experimental paradigms. In addition to providing general models of delusions, an important contribution of this research is that, in demonstrating these biases using delusion neutral material, it has become apparent that these biases represent a more generalized decision-making deficit that extends beyond the content and context of the delusional belief. Recently, the concept of hypersalience of evidence-hypothesis (EVH) matches has been put forward (14, 15) which suggests that the JTC response pattern is caused by increased impact of currently matching evidence, and that BADE response pattern is caused by increased impact of previously matching evidence. However, the issue of which underlying psychological processes may be affected by hypersalience of EVH matches, to allow JTC or BADE to occur in the first place, has not been addressed. We proposed a dual-stream modulation failure (DSMF) model of delusions (16) that provides a potential mechanism that can account for the formation and maintenance of delusions, and the expression of cognitive biases such as JTC and BADE.

Dual-stream information processing models suggest that judgments are the product of a dynamic interaction between reflexive (Stream 1) and reflective (Stream 2) decision-making processes (17). Stream 1 is an automatic form of processing that carries out intuitive, associative analyses of information, and may be guided by habit or emotions (18). Stream 2 is more effortful and analytic, and processes information through logical inferences (18). The more accessible and intuitive Stream 1 may be responsible for most of our daily judgments, with conscious, logical Stream 2 serving to endorse, modify, or reject decisions as appropriate (17, 18).

The DSMF model proposes that the degree to which one stream is favored over another in a specific situation may be determined by two modulating factors: conflict and emotional salience (16). Specifically, a processing conflict may generate a sense of dissonance that may modulate decision-making toward Stream 2, initiating a more thorough consideration of the available evidence, while emotional salience may tip the balance toward the more reflexive and intuitive Stream 1 mode of processing. The diversity of opinions and varying degree of errors displayed by different individuals considering the same information may stem from individual differences in the weighting of these two modulators. However, while suboptimal functioning may contribute to the differences in decision-making that are part of normal human experience, severe aberrations of the modulators, either individually or in tandem, may underlie the formation and maintenance of delusions in schizophrenia. In the DSMF model of delusions, these two cognitive deficits are described as conflict modulation failure (CMF) and accentuated emotional modulation (AEM). CMF suggests that delusions may result from a failure of conflict to adequately modulate decision-making toward Stream 2, diminishing the influence of contradictory evidence on decision-making and increasing the likelihood that erroneous, Stream 1 endorsed beliefs will endure, uncorrected. Psychosis has been associated with an excessive experience of emotion (19, 20), with delusional schizophrenia patients inappropriately attaching increased emotional salience to neutral scenarios (21, 22) or to EVH matches (14). AEM suggests that, in schizophrenia, an aberrantly AEM toward reflexive, Stream 1 processing may further diminish the potentially corrective influence of Stream 2 in instances where Stream 1 interpretations are erroneous.

Predicted neuroanatomical correlates of our dual-stream model of conflict modulation include regions involved in deliberative, logical reasoning, and regions involved in conflict detection or resolution. The deliberative functions of Stream 2 are likely to employ lateral and dorsolateral prefrontal cortex regions. These regions are consistently implicated in a variety of executive reasoning tasks, including the Wisconsin Card Sorting Test (23–27) and the Tower of London test (28, 29), and in both deductive and inductive reasoning tasks (30–33). The conflict detection functions that lead to the engagement of Stream 2 are likely to involve the dorsal anterior cingulate cortex (dACC). Past research has shown that the dACC activates in the presence of cognitive conflict, for example, when encountering incongruent stimuli in the Stroop Task (34–36) and the Go/No-Go Task (37). The conflict modulation arm of the dual-stream modulation model suggests that the presence of cognitive conflict leads to activation of neural regions responsible for mediating a bias toward Stream 2 processing, increasing the likelihood of a response in keeping with the available evidence. This proposal is consistent with a number of theories of dACC function, all of which suggest that, in the presence of cognitive conflict, the dACC signals to other cortical regions that adjustments are needed in order to optimize performance (12, 38–42). These other functional networks work to make adjustments to resolve cognitive conflict, assigning attention to optimize performance. In accordance with this account, dACC activation is highest when an incongruent trial follows a congruent (43, 44), and dACC activation for an incongruent trial predicts increased DLPFC activation in subsequent trials (44). The AEM arm of the model predicts that emotionally salient stimuli would attenuate the activity in this network compared to neutral stimuli.

Previously, we presented behavioral data in support of the CMF arm of the DSMF model (45). Using a simple sentence verification paradigm that put content believability (a Stream 1 judgment) in agreement or in conflict with logical validity (a Stream 2 judgment) we found that the schizophrenia group showed a significantly greater decrease in performance for the conflict condition compared to the non-conflict condition compared to healthy controls. The greater number of erroneous, believability led judgments made by the schizophrenia group when faced with conflict between believability and logical judgment supports the CMF arm of the DSMF model.

The current study uses the same sentence verification paradigm with the addition of neutral and emotionally salient sentences, and in conjunction with fMRI, to replicate and extend our previous findings. The objectives are: (1) replicate our previous findings in support of CMF in schizophrenia. (2) Determine the neurophysiological correlates CMF using functional magnetic resonance imaging. (3) Assess the effects of emotional salience on performance and brain activation to seek support for the AEM arm of the DSMF model. There are three components of the model which give rise to the following, testable hypotheses.

1. The dual-stream processing component of the model predicts that for both groups, the conflict condition will be experienced as more difficult than the non-conflict condition, leading to more errors for the conflict condition than the non-conflict condition.
2. The emotional modulation component predicts that for both groups the emotional stimuli set will lead to more errors for the emotional conflict condition compared to the neutral-conflict condition. The emotional modulation component also predicts that in regions that are activated by the performance of the sentence verification task, the emotional conflict condition will have less activation than the neutral-conflict condition in both groups. The AEM component predicts that these effects will be larger in the patients compared to the controls.
3. The conflict modulation component of the model predicts that for both groups, the conflict condition will result in a greater number of errors and a greater magnitude of activation compared to the non-conflict condition in the network of brain regions that subserve Stream 2 processing. The CMF component of the model predicts that patients will have a greater increase in the number of errors for the conflict condition relative to the non-conflict condition compared to healthy controls. CMF also predicts that patients will have a smaller increase in the magnitude of activation in the identified Stream 2 network for the conflict condition relative to the non-conflict condition compared to healthy controls.

In addition to these hypotheses that are derived from components the DSMF models, we anticipate that, consistent with other studies of cognitive processing in schizophrenia, there will be a general deficit in performance, characterized by a greater number of errors in the schizophrenia group compared to the control group for all conditions.

The primary contrasts of interest for the fMRI data are the group by task interactions. While the main effects between conflict and non-conflict, and the main effects between groups are expected to be quite robust, the group by task interactions are anticipated to be much smaller. To minimize the Type II error that may occur with whole brain correction for multiple comparisons when the effects are anticipated to be small, we will perform a two-stage analysis procedure. In the first stage, a whole brain analysis will be used to identify the network responsible for the primary sentence verification task, with stringent controls for multiple comparisons. In the second stage, the average beta estimates will be extracted for the magnitude of the response within the identified region for each condition and each subject, to test for the interactions of interest within the identified network.

## Materials and Methods

### Participants

Twenty-one participants with schizophrenia and 21 healthy control participants were recruited. All were right-handed, between 20 and 58 years of age, were proficient in English (receiving at least part of their elementary school education and all subsequent education in English), and had normal or corrected-to-normal visual acuity.

Participants in the schizophrenia group were recruited from inpatient psychiatric units at Vancouver General Hospital and the University of British Columbia (UBC) Hospital, affiliated outpatient psychiatric programs, and by advertisement in local newspapers. Patients were diagnosed with schizophrenia or schizoaffective disorder by their hospital or community treating psychiatrist. This diagnosis was confirmed in a separate diagnostic interview conducted by the investigation psychiatrist (ETN). All patients fulfilled the DSM-IV criteria for schizophrenia, though three patients also met the criteria for schizoaffective disorder. Those who met the DSM-IV criteria for substance abuse and dependence, or had a history of serious head injury were excluded from participation in this study. Symptom severity was assessed using the Signs and Symptoms of Psychotic Illness scale [SSPI; (46)], a symptom scale comprising 20 items scored 0–4 according to severity. This scale was administered to all the participants by ETN, a co-developer of the scale, with the mean total symptom score indicating that the patient group was in the moderate range of symptom severity (mean = 9.86, SD = 5.71). As this study was designed to test a model for the formation and maintenance of aberrant belief systems, we preferentially selected for schizophrenia patients with aberrant beliefs. All patients were taking a stable dose of neuroleptic medication, defined as no changes in regular dosages of medication and no requirement for as needed medications in the 4-weeks prior to participation in this study. Twelve participants in this group were receiving one atypical antipsychotic medication (clozapine, olanzapine, risperidone, or quetiapine), one was receiving one typical antipsychotic medication (pipotiazine), and the remaining eight were receiving one of the following combinations of medications: clozapine and lamotrigine; clozapine and valproic acid; risperidone and valproic acid; risperidone and quetiapine; quetiapine, ziprasidone and methotrimeprazine; olanzapine, quetiapine and divalproex sodium; flupentixol and lithium carbonate; loxapine and aripiprazole.

Healthy control participants were recruited through public advertising. In addition to the exclusion criteria for the patients, controls were also excluded if they were currently being treated for a psychiatric condition, had a history of any Axis I diagnosis or had a family history of psychotic illness in a first degree relative. All participants that took part in the study gave written informed consent after a full explanation of the study and the procedures it involved. All experimental procedures were approved by the UBC Clinical Research Ethics Board.

### Materials

Conditional statements were constructed using a single premise (i.e., “If …”) and a single conclusion (i.e., “then …”), with each clause containing a categorical proposition (i.e., all, no, some, some not), e.g., “If no A’s are B’s, then all B’s are A’s.” Both internal and external validity were considered when constructing the conditional statements. Internal validity refers to the logical validity of the whole statement; a deliberative, Stream 2 assessment of whether or not the conclusion logically follows the premise. Internal validity is determined by the specific pairing of categorical propositions used, not the subject matter. “If no A’s are B’s, then all B’s are A’s,” is logically invalid regardless of whether the subject pair A and B refers to bank tellers and women (“If no bank tellers are women, then all women are bank tellers”) or criminals and rapists (“If no criminals are rapists, then all rapists are criminals”) as it is constructed using the categorical proposition pair, “no … all.” External validity is the validity of the conclusion independent from the premise. It can be considered as the “believability” of the conclusion; an associative, Stream 1 assessment of the consistency between the conclusion and the participant’s semantic knowledge base. External validity is a function of both the subject and its categorical proposition, e.g., “all women are bank tellers,” is externally invalid, while, “all rapists are criminals,” is externally valid. The dissociation between internal validity and external validity allows for the creation of conditional statements where internal validity and external validity either conflict (beliefs do not match logical validity) or agree (i.e., beliefs match logical validity).

Forty neutral stimuli were created from 20 subject pairs that all participants could be reasonably expected to be familiar with in terms of believability judgments. Each item was used to create both a conflict and a non-conflict statement.

An emotionally salient stimuli set was selected using a pilot questionnaire of 80 emotionally salient statements that was given to 15 healthy control participants. The statements were of a form that could be readily translated into two-part conditional sentences (e.g., “Rapists are criminals” could become “If no criminals are rapists, then no rapists are criminals”). Participants rated each statement for “valence” (a seven point scale ranging from “Strongly Disagree” to “Strongly Agree”) and “arousal” (a five point scale ranging from “low” to “high” arousal). Research using the Self-Assessment Manikin (47, 48) has shown that the most emotionally salient stimuli are those that are rated at the extremes for both valence and arousal, with increases in ratings on one scale generally corresponding to increases in the ratings for the other. For this study we operationalized “emotional salience” as the sum of the ratings for valence and arousal, with the most emotionally salient items being considered those with the highest summed score. The 20 items with the highest emotional salience score across all participants were selected to create 40 stimuli, with each item being used to create a conflict and a non-conflict statement.

### Procedure

Prior to participation in the task, all participants were screened for safety for high-field MRI in accordance with the guidelines of the UBC High-Field MRI Center, provided informed consent, and completed an assessment battery consisting of the National Adult Reading Test (NART) (49) as a proxy for premorbid IQ, and the Ammons Quick Test [QUICK; (50)] as a proxy for current IQ. A training session was given on a laptop computer to familiarize participants with the conditional reasoning task and its timing. Instructions were given making it clear that determinations of logical validity related to the internal validity of the statements. When it was clear that the task was understood (operationalized as six of eight neutral exemplars being answered correctly), the experimental phase was initiated.

Participants underwent fMRI scanning while determining the logical validity of conditional sentences constructed as described in detail above. Responses were given by pressing one of two buttons to indicate whether conditional sentences were logically valid or logically invalid. Each trial began with a 3-s presentation of the “If” clause, followed by presentation of the whole “If … then” statement for a maximum of 9 s further. When a response was given, via button press, the statement was replaced with a crosshair for 3, 4, or 5 s. Trials were separated by crosshair presentation regardless of whether a response was given or not.

The study comprised four runs of 20 conditional sentences for a total of 80 conditional sentences. Forty were neutral stimuli and 40 emotionally salient. Of each 40, 20 were conflict stimuli and 20 were non-conflict stimuli. Each of the four runs was balanced for conflict status, salience, and internal validity. Five null periods of 15, 16, or 17 s occurred randomly across each run. The inclusion of null events provides trial-free periods, allowing baseline levels of activation to be attained (51).

### Image acquisition and processing

Echo-planar images were collected on a Philips Achieva 3.0-T scanner, equipped with a SENSE coil. Conventional spin-echo T1-weighted sagittal localizers were used to view head position and to graphically prescribe the functional image volumes. Functional image volumes sensitive to the blood-oxygen-level-dependent (BOLD) contrast signal were collected with a gradient echo sequence (TR/TE 2000/30 ms, 90°flip angle, FOV 216 mm × 143 mm × 240 mm (AP, FH, RL), 3.00 mm slice thickness, 1 mm slice gap, and 36 axial slices). Functional images were reconstructed offline. Statistical Parametric Mapping software (SPM5, Wellcome Institute of Cognitive Neurology) was used for image reorientation, realignment, normalization into Montreal Neurological Institute space, and smoothing with a Gaussian kernel (8 mm full width at half maximum) to compensate for inter-participant anatomical differences and optimize the signal-to-noise ratio. Images and movement parameters were screened for potential movement artifacts prior to data analysis. No excessive head movement (>2 mm) was observed in any of the participants.

### Data analysis

Between groups analyses of demographic and IQ measures were carried out using two sample *t*-tests, with a Chi-Square goodness-of-fit test for sex.

In addition to the specific *t*-test corresponding to our *a priori* hypotheses, the behavioral data (response accuracy) was analyzed using a 2 × 2 × 2 analyses of variance (ANOVA) (SPSS 12.0 for Windows, SSPS Inc., Chicago, IL, USA). A trial was recorded as an error if either an incorrect response was given or no response was given before the beginning of the next trial. The independent variables were group (healthy controls and participants diagnosed with schizophrenia), conflict status (conflict and non-conflict conditionals), and salience (neutral and emotionally salient). The dependent variable was response accuracy (percentage of correct responses). Additional *t*-tests were carried out to determine whether the difference between conditions (conflict minus non-conflict) was significantly different between groups for both neutral and emotionally salient stimuli. Statistical tests for behavioral and functional imaging data were one-tailed, reflecting the directional nature of our hypotheses.

**Figure 1:**
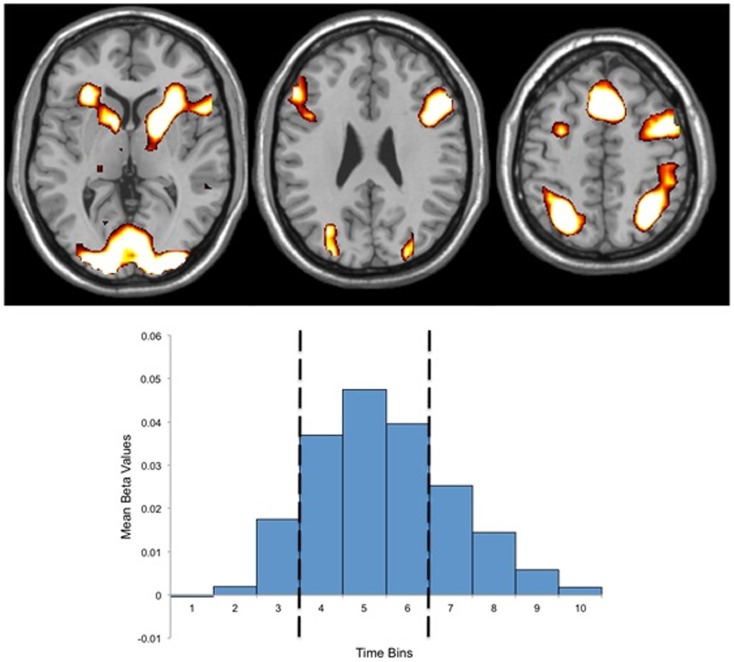
***Above:* regions of activation for the mask of task related activity**. *Below:* Finite Impulse Response (FIR) basis set (10 time bins) for the mask of task related activity, indicating the peak hemodynamic response occurring during time bin five, and the three time bins used for beta estimate extraction (bins four through six). Both activations and the FIR basis set present data for both groups.

fMRI data analysis comprised three stages: (1) the task related BOLD response was estimated using a set of 10 finite impulse response (FIR) functions (52, 53) corresponding to the 10 repetition times (20 s) immediately following the presentation of each “If …” statement. FIR models make no *a priori* assumptions with regards to the shape and time course of hemodynamic response functions, and thus, avoid errors associated with ill-fitting canonical models (54). (2) The beta estimates for the FIR models were brought forward for a second level analysis to identify the networks of brain regions that showed significant activity during task performance (*t* = 7.94, *p* = 0.00001, corrected for multiple comparisons). To avoid a group or condition bias, this analysis was performed on all participants (both patients and controls) for all conditions combined. (3) The beta estimates for each FIR function for each voxel within the identified network were extracted for each participant for each stimulus type. The mean beta estimates for time bins four to six were calculated for each subject and each stimulus type and used as dependent variables to test our specific hypotheses as well as in a 2 × 2 × 2 (group by conflict status by salience) ANOVAs. Time bins four through six were chosen to maximize the signal-to-noise ratio as a review of the observed BOLD response indicated that the peak BOLD response occurred during this period for both groups and both conditions (Figure 1).

Bivariate Pearson correlation coefficients were calculated using performance on the conflict condition and mean beta values for the conflict condition to test whether there was a positive correlation between success on the conflict condition and amount of activity for the whole task activation network.

## Results

### Sociodemographic and psychopathological characteristics

The mean duration of illness for the patient group was 15.67 years (SD 10.89 years) (Table 1). The mean total score on the SSPI was 9.86 (SD 5.71). Sixteen of the 21 patients endorsed aberrant beliefs; of these 16, five had severe delusions (SSPI delusions score = 4), six had definite delusions (SSPI delusions score = 3), and five exhibited unrealistic beliefs bordering on delusions (SSPI delusions score of 1 or 2).

**Table 1:** **Sociodemographic and psychopathological group characteristics**.

	Healthy controls (*n* = 21)	Schizophrenia (*n* = 21)	*t*-Value	*p*-Value
Age (years)	33.86 (10.76)	34.67 (11.25)	−0.238	0.813
Sex (M:F)	14:7	14:7	0.000	1.000
Education (years)	15.24 (2.23)	13.76 (2.55)	1.996	0.053
NART (IQ)	118.86 (4.88)	119 (5.34)	−0.090	0.928
QUICK (IQ)	109.9 (10.47)	105.6 (14.2)	1.109	0.274
Illness duration (years)	n/a	15.67 (10.89)		
SSPI (delusions)	n/a	2.14 (1.56)		
SSPI (total)	n/a	9.86 (5.71)		

*Mean values, with SD in parentheses are presented*.

There were no significant between groups differences in age [*t*(40) = −0.238, *p* = 0.81], sex [χ^2^ (1, *N* = 40) = 0.00, *p* = 1.00], QUICK scores [*t*(40) = 1.109, *p* = 0.27], or NART scores [*t*(40) = 0.09, *p* = 0.93]. There was a significant between groups difference for years of education [*t*(40) = 1.996, *p* = 0.05]. However, there were no significant correlations between years of education and performance (percentage answered correctly) for either group [Controls: *r*(21) = −0.093, *p* = 0.688; Sz: *r*(21) = 0.133, *p* = 0.566].

### Behavioral results

#### ANOVA main effects and interactions: behavioral data

**Figure 2:**
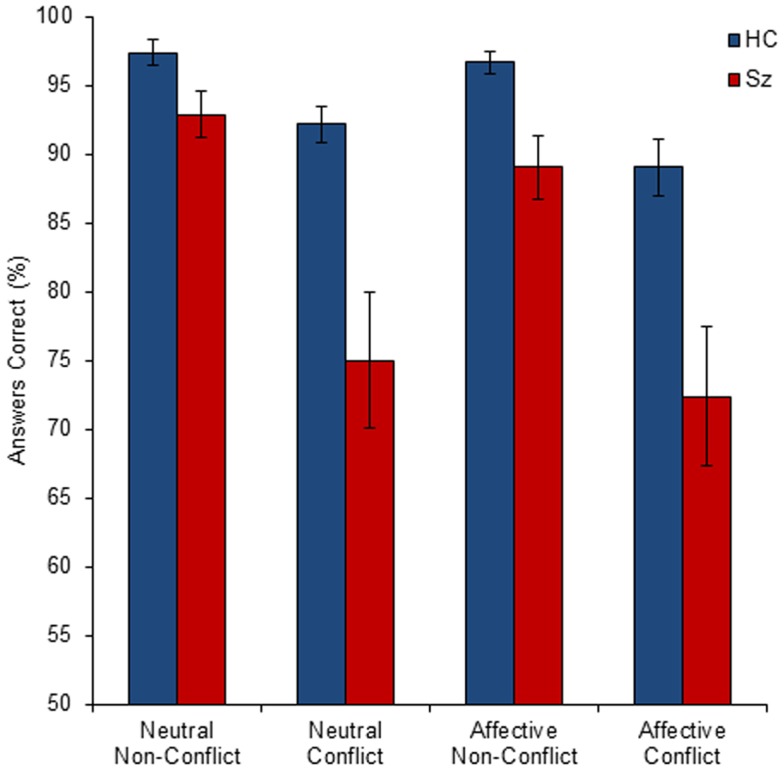
**The mean percentage of correct responses by group (HC, healthy control; Sz, schizophrenia), conflict status (conflict and non-conflict), and salience (affective and neutral)**. Error bars show standard error of the mean (SEM). All between-group comparisons were statistically significant.

The mean group performance (percentage of conditional statements answered correctly) for all conditions is shown in Figure 2. The 2 × 2 × 2 (group by salience by conflict status) ANOVA indicated highly significant main effects of group (*F*_1, 160_ = 32.25, *p* < 0.001) and conflict status (*F*_1, 160_ = 32.25, *p* < 0.001), with a significant interaction of group by conflict status (*F*_1, 160_ = 7.17, *p* < 0.01). There was no significant main effect of salience (*p* = 0.21) and no significant interactions of group by salience (*p* = 0.75), salience by conflict status (*p* = 0.88), or group by salience by conflict status (*p* = 0.66).

#### *T*-tests for specified *a priori* hypotheses: behavioral data

Consistent with the general deficits across wide cognitive domains reported for schizophrenia, the control group performed significantly better than the schizophrenia group for all four conditions: neutral [*t*(20) = 3.54, *p* < 0.001], affective [*t*(20) = 3.45, *p* < 0.001], non-conflict [*t*(20) = 2.96, *p* < 0.005], conflict [*t*(20) = 3.33, *p* < 0.001].

Consistent with dual-stream processing, both groups performed better for the non-conflict condition compared to the conflict condition: patients [*t*(20) = 4.18, *p* = 0.0002], controls [*t*(20) = 4.40, *p* = 0.0001].

Contrary to the emotional modulation model there were no significant differences between (affective-conflict errors – neutral-conflict errors) for either group: patients [*t*(20) = 0.35, *p* = 0.36], controls [*t*(20) = 1.30, *p* = 0.11].

Contrary to the AEM model, patients did not show a greater difference for affective-conflict errors minus neutral-conflict errors compared to controls: patient difference = 2.6 errors, control difference = 3.1 errors [*t*(20) = 0.55, *p* = 0.29].

Consistent with CMF, the patients had a significantly greater difference in the number of errors for (conflict errors – non-conflict errors) compared to controls: patients made 17.3 more errors for the conflict condition than the non-conflict condition compared to controls, who only made 6.4 more errors [*t*(20) = 2.52, *p* = 0.01].

### Functional imaging results

Performance of the conditional sentence task significantly activated a network of 3664 voxels (Figure 1). The location and magnitude of local peaks as well as the extent of the subclusters that comprise this network are listed in Table 2. The mean magnitude of the activation within the network for each condition and each group are shown in Table 3.

**Table 2:** **Localization of activations for task related activation clusters [voxels showing significant activity (*t* = 7.94, *p* = 0.00001, family wise error (FWE) correction for multiple comparisons), irrespective of group, and across both conditions]**.

Cluster name	Peak MNI coordinates (*x*, *y*, *z*)	Voxels	*t*	*p*
Occipital cortex/parietal lobules	24, −100, −4	2160	20.99	0.000
Dorsal anterior cingulate cortex	0, 16, 48	290	16.54	0.000
Prefrontal cortex/striatum	−20, 8, −4	803	14.26	0.000
Insula/striatum	32, 24, −4	292	12.76	0.000
Prefrontal cortex	56, 28, 24	76	12.16	0.000
Precentral cortex	32, 0, 52	31	10.4	0.000
Hippocampus	24, −28, −4	9	9.47	0.000
Inferior frontal operculum	60, 16, 4	1	8.45	0.000
Hippocampus	−24, −32, −4	1	8.26	0.000
Fusiform gyrus	44, −28, −16	1	8.12	0.000

**Table 3:** **Mean beta estimates of the magnitude of activation ± the standard error of the mean for each condition for patients and controls**.

Condition	Controls	Patients
Neutral non-conflict	0.036 ± 0.003	0.033 ± 0.003
Neutral conflict	0.047 ± 0.003	0.039 ± 0.003
Affective non-conflict	0.047 ± 0.003	0.041 ± 0.003
Affective conflict	0.049 ± 0.003	0.038 ± 0.004

#### ANOVA main effects and interactions: fMRI data

The 2 × 2 × 2 (group by salience by conflict status) ANOVA of mean beta estimates (time bins four through six) showed a significant main effect of group, with greater activity for the healthy control group than schizophrenia group [controls = 0.045 (SD = 0.015), patients = 0.038 (SD = 0.015); (*F*_1, 160_) = 10.24, *p* < 0.01], and a main effect of salience, with greater activity for affective compared to neutral stimuli [neutral = 0.039 (SD = 0.015), affective = 0.044 (SD = 0.016); (*F*_1, 160_) = 4.93, *p* < 0.05]. There was a trend toward significance for conflict status, with greater activity for conflict stimuli than non-conflict [conflict = 0.043 (SD = 0.016), non-conflict = 0.039 (SD = 0.015); (*F*_1,160_) = 3.37, *p* = 0.068], and a trend for the interaction between conflict status and salience [(*F*_1, 160_) = 3.66, *p* = 0.057].

#### *T*-tests for specified *a priori* hypotheses: fMRI data

Consistent with decreased performance being associated with decreased activity across diverse cognitive tasks in schizophrenia, patients showed a general decrease in activity in the identified network compared to patients for all four conditions combined. The mean beta estimate of activity for the controls was 0.044 compared to 0.038 for the patients [*t*(20) = 1.75, *p* = 0.037].

In contrast to the predicted decrease in activity for affective-conflict stimuli compared to neutral-conflict stimuli (outlined in the emotional modulation component of the model), there was no significant difference in the activation between these conditions in either group controls affective-conflict beta = 0.049, neutral-conflict beta = 0.046, *t*(20) = 0.53, *p* = 0.60; patients affective-conflict beta = 0.038, neutral-conflict beta = 0.039, [*t*(20) = 0.50, *p* = 0.62]. The greater decrement in activation for affective-conflict stimuli in patients compared to controls predicted by the AEM arm of the model was not observed [difference in controls = −0.002, difference in patients = 0.001, *t*(20) = 1.35, *p* = 0.19].

Consistent with conflict modulation, both groups showed greater activation for conflict stimuli compared to non-conflict stimuli [controls: conflict = 0.048, non-conflict 0.042, *t*(20) = 4.78, *p* < 0.001; patients: conflict = 0.039, non-conflict 0.037, *t*(20) = 2.1, *p* = 0.05]. Consistent with CMF, controls showed a significantly greater increase in activity for the conflict condition relative to the non-conflict condition than did patients [control difference (conflict minus non-conflict) = 0.006, patient difference = 0.002, *t*(20) = 2.42, *p* = 0.01].

**Figure 3:**
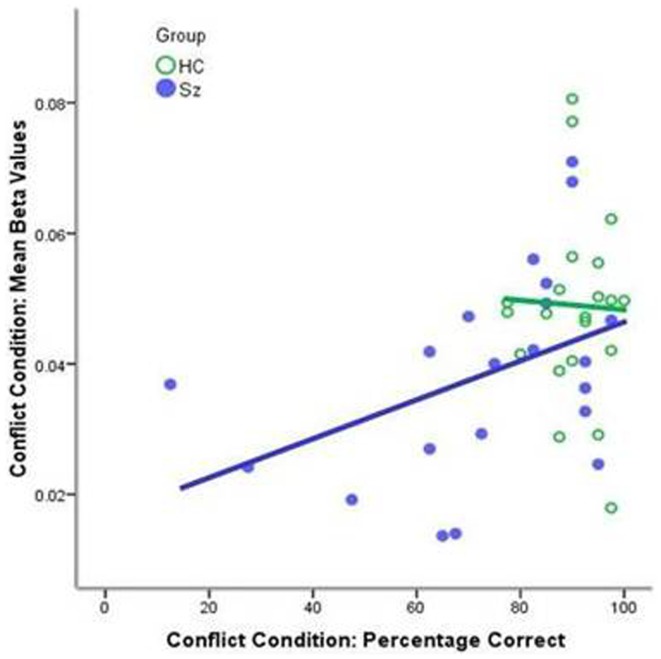
**Correlations between performance on the conflict condition and the mean beta values for the conflict condition for the whole mask**. The schizophrenia group showed a significant correlation [*r*(21) = 0.422; *p* < 0.05, blue line]; the healthy control group displayed uniformly high level of performance [*r*(21) = −0.04; *p* = 0.432, green line].

The schizophrenia group showed significant correlations between performance on the conflict condition and BOLD activity associated with the conflict condition for the entire network [*r*(21) = 0.422; *p* < 0.05; Figure 3]. There was no significant correlation for the healthy control group [*r*(21) = −0.04; *p* = 0.432].

## Discussion

The DSMF model of delusions suggests two processing aberrations that may contribute to delusion formation and maintenance in schizophrenia: a failure of cognitive conflict to adequately increase deliberative, Stream 2 processing (CMF) and an AEM away from Stream 2 and toward non-deliberative, Stream 1 processing. These modulation deficits may occur in tandem or separately, leading to an under-recruitment of Stream 2 processing and/or an increase in (or relative failure to suppress) Stream 1 processing. This creates a cognitive environment were erroneous, intuitive beliefs are more likely to be endorsed, and then endure, despite minimal evidential support.

The data presented replicate and extend previous findings in support of the conflict arm of the DSMF model. The interference of believability with assessments of logical validity is a well-documented phenomenon in healthy controls (32, 55–57). This replication is neither novel nor surprising in that the experience of dissonance resulting from such conflicts are common occurrences in everyday life. Single stream processors, such as computers, attempting reasoning tasks using logic or set theory would not require additional time or resources to solve the task if there was a conflict between the logical solution and “knowledge” residing on its hard drive. The proposed dual-stream modulation model provides a potential cognitive mechanism that accounts for this commonly experienced interference in humans. CMF predicts that this effect will be exaggerated in people diagnosed with schizophrenia (16, 45). As in our previous study (45), the schizophrenia group exhibited a further deficit in performance for the conflict condition that went above and beyond the general performance deficit reflected in the group difference for the non-conflict condition.

Similar to the previous study (45) the control group had more years of education than the schizophrenic group. The difference in years of education between groups is likely a consequence of illness interrupting education in the schizophrenia group, such that the well-established cognitive deficits associated with schizophrenia may have directly contributed to the differences in years of education. Matching the samples on years of education would require one of the groups to be non-representative (i.e., either highly educated patients or poorly educated controls), and may not be an appropriate strategy for the schizophrenia group if years of education is a dependent variable for this group. However, in the syllogism literature, years of education and IQ do correlate with performance in healthy controls, and so the difference in years of education cannot be ruled out as a contributing factor to the group difference in performance in the current study where the schizophrenia group performed worse than healthy controls for both conditions. While years of education may improve general formal logical reasoning skills, it is less clear how years of education would correspond to a specific enhancement in the ability to inhibit belief-biased responding in the conflict condition. Belief-incongruence is considered the source of decreased performance between conditions where syllogisms are formally identical, but populated with content varying in believability. The greater drop in performance for the schizophrenia group for conflict compared to non-conflict conditions suggests a greater susceptibility to the belief-bias effect, in addition to a general performance deficit. This finding is consistent with CMF.

This study extends previous behavioral findings by providing neurophysiological data consistent with the processing differences predicted by CMF. As expected for a cognitively demanding task, participants demonstrated a central executive network (CEN) (58, 59)/task-positive functional network (60)/multiple demands network (61) pattern of activation, consistent with other studies utilizing deductive reasoning paradigms [e.g., (62–64)], and including nodes associated with both conflict processing (dACC) and deliberative reasoning (L/DLPFC). Consistent with conflict modulation, namely, an increase in deliberative processing when presented with a conflict stimulus (12, 38–42, 65), the healthy control group showed a significant increase in activity in this network for the conflict condition compared to the non-conflict. A significantly smaller increase was observed in the schizophrenia group, consistent with research demonstrating attenuated dACC activity for schizophrenia patients in response to conflict stimuli in the Stroop task (35) and for error commission in the Go/No-Go task (66). Within the conflict arm of the model, the increase in activity for the conflict condition may represent enhanced engagement of Stream 2 processes when faced with cognitive conflict. The increase in activity for the conflict condition is not due to increase complexity of the logic component of the task. The logic component of the task is exactly the same for both conflict and non-conflict conditions. The observed increase in activity is due to the presence of the conflict between Stream 1 and Stream 2 processes. The failure of the schizophrenia group to enhance activity in this network to the same degree as healthy controls may be the physiological basis for the greater decrease in performance for the conflict condition displayed by the schizophrenia group. The interpretation that an increase in CEN activity when confronted with cognitive conflict corresponds to an increase in Stream 2 processing that reduces the likelihood of believability led errors is further supported by the positive correlation between the magnitude of activity in this network and task performance for the conflict condition in the schizophrenia group. The absence of a significant correlation in the healthy controls reflects the high level of performance or ceiling effect in this group.

This is the first study investigating the AEM arm of the DSMF model. AEM predicts that emotions modulate decision-making toward Stream 1 in both groups and that this modulator effect is accentuated in the schizophrenia group. Emotional modulation should lead to more errors for affective-conflict condition compared to neutral-conflict conditions in both schizophrenia and control groups. AEM predicts that this difference would be larger for the schizophrenia group than the control group. The model further predicts that emotional modulation would attenuate the neurophysiological network that subserves Stream 2 processing for the affect-conflict condition relative to the neutral-conflict condition for both groups and that this attenuation would be accentuated in the schizophrenia group. In this study, emotional salience did not lead to further decreased performance for the conflict condition in either group. Contrary to our predictions of decreased activity in Stream 2 networks, both groups showed increased activity in the identified network for the emotional-salient stimuli compared to neutral stimuli.

These results suggest either that the “emotional salience leads to decreased performance for the conflict condition” arm of the AEM model is incorrect, or the stimuli set was not suitable to test emotional modulation. The affective stimuli set in our study was selected in piloting via subjective ratings of arousal and salience. It is possible that these stimuli were not sufficiently personally salient or arousing enough to induce the anticipated effect. A sufficient threshold of intensity may need to be exceeded before the effect will be seen, particularly as increased cognitive demands are associated with decreases in activity in regions of the brain associated with processing affect [e.g., (67, 68)].

## Conclusion

The current results provide further behavioral support for both conflict modulation in healthy controls and CMF in schizophrenia. Additionally, this study indicates that conflict modulation toward Stream 2 processing may be associated with an increase in CEN activity, which includes regions previously identified as involved in conflict detection and deliberative processing (i.e., dACC and frontal cortex). CMF in schizophrenia may be the result of a failure to adequately engage this network, increasing the likelihood of erroneous judgments when faced with belief-logic conflicts.

## Conflict of Interest Statement

The authors declare that the research was conducted in the absence of any commercial or financial relationships that could be construed as a potential conflict of interest.
